# A novel rat model for cerebral venous sinus thrombosis: verification of similarity to human disease via clinical analysis and experimental validation

**DOI:** 10.1186/s12967-022-03374-y

**Published:** 2022-04-11

**Authors:** Shuwen Mu, Yinghong Lin, Yongjun Xu, Xianqing Wei, Zihuan Zeng, Kunzhe Lin, Linghua Zhu, Qinghong Liu, Xingfeng Qi, Liangfeng Wei, Shengxiang Liang, Shousen Wang

**Affiliations:** 1 Department of Neurosurgery, Fuzong Clinical Medical College of Fujian Medical University, Fuzhou, 350025 China; 2 Fujian University of Traditional Chinese Medicine, Fuzhou, 350122 China; 3 Laboratory of Basic Medicine, 900th Hospital, Fuzong Clinical Medical College of Fujian Medical University, Fuzhou, 350025 China; 4 College of Materials Science and Engineering, Shenyang University of Technology, Shenyang, 110870 China; 5 Department of Neurosurgery, 900th Hospital, Fuzong Clinical Medical College of Fujian Medical University, Fuzhou, 350025 Fujian China; 6 Department of Neurosurgery, Affiliated Fuzhou First Hospital of Fujian Medical University, Fuzhou, 350009 China; 7 Department of Pathology, 900th Hospital, Fuzong Clinical Medical College of Fujian Medical University, Fuzhou, 350025 China; 8 National-Local Joint Engineering Research Center of Rehabilitation Medicine Technology, Fujian University of Traditional Chinese Medicine, Fuzhou, 350122 Fujian China; 9 Rehabilitation Industry Institute, Fujian University of Traditional Chinese Medicine, Fuzhou, 350122 China

**Keywords:** Cerebral venous sinus thrombosis, Animal model, Pathophysiology, Vasogenic oedema

## Abstract

**Background:**

Cerebral venous sinus thrombosis (CVST) is a rare neurovascular disorder with highly variable manifestations and clinical courses. Animal models properly matched to the clinical form of CVST are necessary for elucidating the pathophysiology of the disease. In this study, we aimed to establish a rat model that accurately recapitulates the clinical features of CVST in human patients.

**Methods:**

This study consisted of a clinical analysis and animal experiments. Clinical data for two centres obtained between January 2016 and May 2021 were collected and analysed retrospectively. In addition, a Sprague–Dawley rat model of CVST was established by inserting a water-swellable rubber device into the superior sagittal sinus, following which imaging, histological, haematological, and behavioural tests were used to investigate pathophysiological changes. Principal component analysis and hierarchical clustering heatmaps were used to evaluate the similarity between the animal models and human patients.

**Results:**

The imaging results revealed the possibility of vasogenic oedema in animal models. Haematological analysis indicated an inflammatory and hypercoagulable state. These findings were mostly matched with the retrospective clinical data. Pathological and serological tests further revealed brain parenchymal damage related to CVST in animal models.

**Conclusions:**

We successfully established a stable and reproducible rat model of CVST. The high similarity between clinical patients and animal models was verified via cluster analysis. This model may be useful for the study of CVST pathophysiology and potential therapies.

**Supplementary Information:**

The online version contains supplementary material available at 10.1186/s12967-022-03374-y.

## Background

Cerebral venous sinus thrombosis (CVST) is a rare neurovascular disorder accounting for approximately 0.5% of strokes, occurring more frequently in young adults [1]. Its clinical manifestations lack specificity, including headaches, epileptic seizures, different degrees of neurological deficits, and even coma or death in severe cases [2, 3]. A recent clinical study reported that the incidence of CVST has been historically underestimated [4]. In addition, the two latest clinical studies have highlighted the need to address vaccination-associated CVST given the global pandemic of COVID-19 [5, 6]. As few patients have any symptoms at the initial stage and many uncontrollable factors are involved during disease development, clinical research tends to be severely hampered [7]; therefore, the pathophysiology of CVST remains to be elucidated.

Animal models that appropriately recapitulate the clinical symptoms are essential for understanding CVST pathophysiology. Over the past few years, several types of CVST models have been established, including those generated via superior sagittal sinus (SSS) ligation [8, 9], injection of thrombi or procoagulants [10–12], thrombus models induced using ferric chloride, and SSS occlusion via a self-made plug [13, 14]. Despite the various modelling methods, each model has explained the pathophysiology of CVST from a different perspective. Moreover, most models require invasive operation, which can lead to iatrogenic injury of the brain parenchyma. In addition, the risk of uncontrollable spontaneous recanalisation and lack of positive findings highlight the limitations of current therapeutic strategies [15]. Therefore, establishing an animal model that appropriately captures clinical features in human patients is essential for developing standardised treatment strategies.

In the present study, we aimed to establish a rat model that accurately recapitulates the clinical features of CVST in human patients by inserting a water-swellable rubber implant into the SSS, following which we investigated the associated pathophysiological changes using imaging, histological, and haematological tests. Neurological function was also examined using behavioural tests. The clinical data of patients with CVST were collected for retrospective analysis and used to verify the similarity between models and clinical features of CVST.

## Methods

### Clinical data

We retrospectively analysed clinical data obtained at two centres (900th Hospital and Affiliated Fuzhou First Hospital of Fujian Medical University) between January 2016 and May 2021. A total of 48 adult patients (age > 18 years) with definite diagnoses of acute or subacute CVST were selected from the database [16]. Exclusion criteria were as follows: (a) insufficient patient information, including missing data for imaging, laboratory indicators (blood routine and coagulation tests), or CVST severity (Glasgow Coma Scale [GCS], National Institutes of Health Stroke Scale [NIHSS], and modified Rankin Scale [mRS] scores); (b) CVST induced by other intracranial injuries, such as intracranial tumours, traumatic brain injury, brain abscess, and meningitis. A control group of healthy adults within the same age range was included for comparison.

Patients were followed up via telephone, and a total of 34 patients with valid information were included. Outcomes were assessed based on the mRS, with an mRS ≤ 1 defined as good recovery [17]. Mental status was assessed using the Beck Anxiety Inventory (BAI), Beck Depression Inventory (BDI), and Hamilton Depression Scale (HAMD). The BAI was used to screen patients with anxiety [18], while the BDI and HAMD were used to screen patients with depression [19, 20].

All procedures performed in studies involving human participants were in accordance with the ethical standards of the institutional and/or national research committee and with the 1964 Helsinki Declaration and its later amendments or comparable ethical standards. This study was approved by the local Ethics Committee (Fuzhou, China) of the two centres, and the requirement for informed consent was waived due to the retrospective study design.

### Animal preparation

Male Sprague–Dawley rats (280–300 g, age: 7–8 w, Animal Experimental Center of Fujian Medical University, Fuzhou, China) were used in this study. This animal experiment was in accordance with the Guide for the Care and Use of Laboratory Animals (Institute for Laboratory Animal Research, National Research Council. Washington, DC: National Academy Press, 1996) and was approved by the Fujian Medical University Ethics Committee (No. 2020-051). Animals were maintained in a standard environment of 20–22 °C under a 12-h light/12-h dark cycle, with free access to food and water. A total of 96 rats were randomly divided into two groups: a sham-operated (sham) group (n = 48) and a CVST model group (n = 48). In each group, rats were prepared as follows: n = 8 for MRI, n = 8 for intracranial pressure (ICP) monitoring, n = 8 for routine blood and plasma coagulation tests, n = 8 for histological and serological examination, n = 4 for transmission electron microscopy (TEM), and n = 12 for behavioural tests.

### Production of embolism materials

We chose water-swellable rubber (PZ-250, Hongji Rubber Co., Hebei, China) as the embolic material. The parameters for the rubber device were obtained from multiple measurements of the rat SSS, which were divided by the expansion ratio of the water-swellable rubber (total length: 22 mm; anterior diameter: 1.2 mm; posterior diameter: 0.8 mm) (Additional file 1: Fig. S1a). Next, we collected venous blood from rats to measure the expansion ratio of the material in vitro (Additional file 1: Fig. S1b–d). The rubber was sterilised using ultraviolet light before modelling.

### Model establishment

**Fig. 1:**
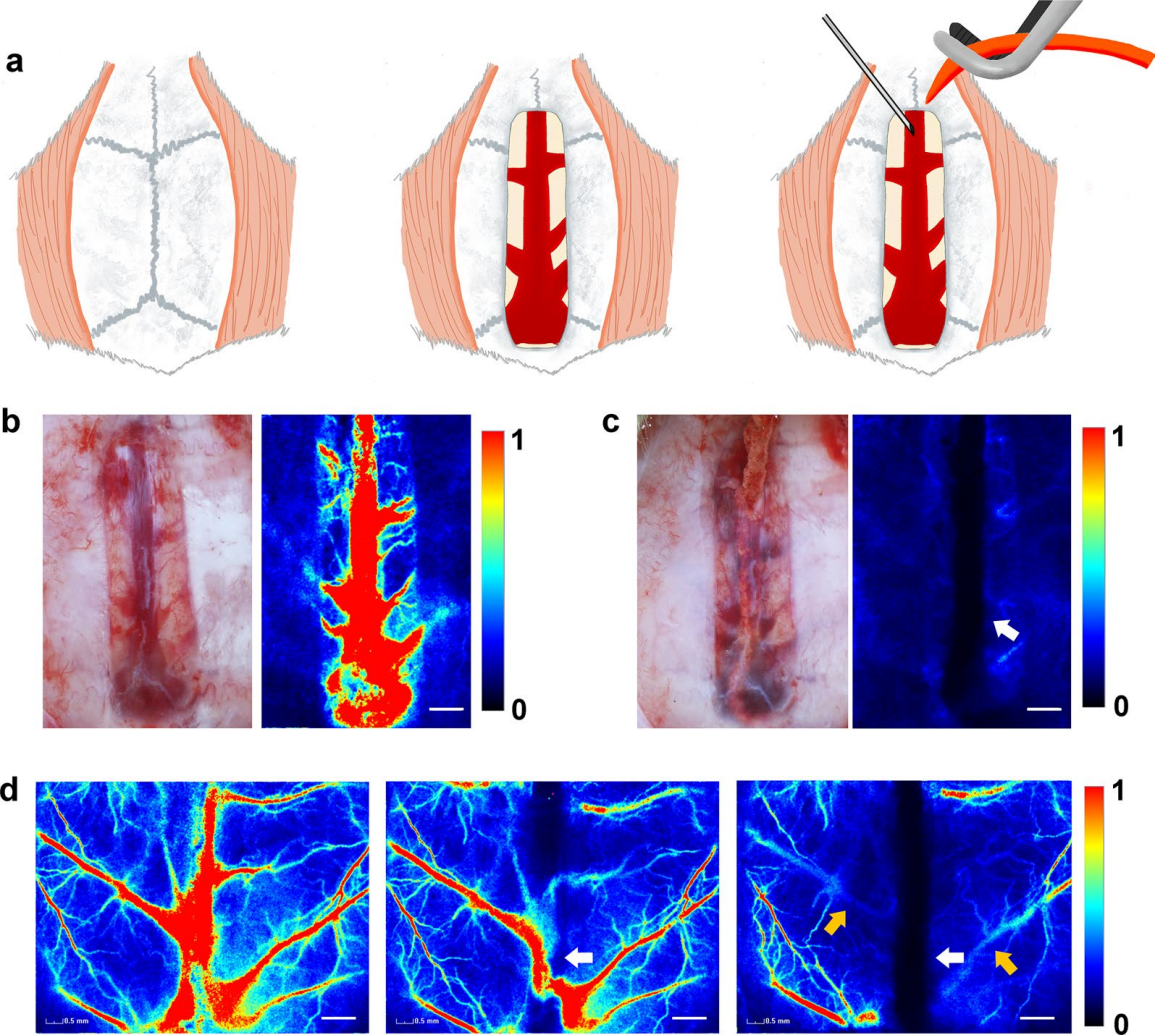
Procedure for establishing the rat model. **a** Modelling process diagram. **b** The bone window is opened to expose the SSS. **c** Water-swellable rubber effectively results in SSS occlusion on the first day (white arrow). **d** Regions of interest obtained by thinning of the skull to monitor superficial cerebral blood flow; images show partial disappearance of SSS blood flow following insertion of the rubber implant (white arrows). Near-complete disappearance and a partial decrease in bridging veins were observed on the first day after modelling (yellow arrows). Scar bar = 1 mm. SSS: superior sagittal sinus

For each rat, anaesthesia was induced with 4% isoflurane delivered via a small-animal anaesthesia machine (RWD Science Co., Shenzhen, China) and maintained with 2% isoflurane at 0.5 L/min. The rat was then fixed in the prone position using a brain stereotaxic apparatus. The respiratory rate was stable at 40–60 per min, and body temperature was stabilised at 37 ± 0.5 °C using a heating pad. A 20-mm midline skin incision was made, following which a cranial window was created around the sagittal sinus using a high-speed dental drill under an operating microscope. The drill tip was repeatedly cooled with normal saline to prevent heat injury during the operation. In the model group, the SSS was carefully punctured, and the rubber was promptly inserted centripetally until the posterior wall of the sinus confluence, following which the excess part of the posterior area was sectioned (Fig. 1a). The operation field was flushed with normal saline several times, and the skin incision was sutured. The sham group was not subjected to rubber insertion. The rats were kept warm until waking and placed in a single cage with food and water available.

### Laser-speckle contrast imaging (LSCI)

The LSCI system (Wuhan SIM Opto-technology Co., Wuhan, China) was composed of an Olympus ZS61 microscope, continuous-wavelength laser source, charge-coupled-device camera, and a computer. The observation region was illuminated using a laser light source. Speckle signals (exposure time = 10 ms) were continuously collected by the camera and then transmitted to a computer for analysis [21]. To avoid skull impediments to LSCI observation, the skull in the observation region (15 mm × 10 mm) was thinned until the blood vessels were clearly visible.

### MRI

The rats underwent scanning using a small-animal MRI system (9.4T; Bruker Medical GmbH, Ettlingen, Germany) on postoperative days 1 and 7. After successful anaesthesia, each rat was fixed in the prone position on the MRI scanning bed, and T2-weighted imaging (T2WI), diffusion-weighted imaging (DWI), and magnetic resonance angiography (MRA) scanning were performed after determining the brain position. T2WI parameters were set as follows: repetition time (TR) = 3000 ms, echo time (TE) = 33 ms, number of excitations (NEX) = 2, slices = 28, slice thickness = 0.8 mm, field-of-view (FOV) = 30 × 30 mm^2^, matrix size = 256 × 256. DWI parameters were set as follows: TR = 2500 ms, TE = 17.5 ms, slices = 28, slice thickness = 0.8 mm, FOV = 30 × 30 mm^2^, matrix size = 128 × 128. The 2D-time of flight method was adopted for MRA, and the parameters were set as follows: TR = 12 ms, TE = 1.9 ms, NEX = 2, slices = 150, slice thickness = 0.2 mm, FOV = 30 × 30 mm^2^, matrix size = 256 × 256, flip angle = 80°.

To extract the apparent diffusion coefficient (ADC), we placed a region of interest (ROI) on the abnormal-intensity area on the DWI while avoiding hematomas, as described in a previous study [22]. All image data were analysed using ITK-SNAP (version 3.8.0).

### ICP monitoring

ICP was measured on the first and seventh days after modelling as described in a previous study [23]. A cranial hole was drilled approximately 3 mm from the left anterolateral side of the coronal suture, and an ICP-monitoring probe was placed with an external ICP monitor (Johnson & Johnson inc., New Brunswick, USA).

### Routine blood and plasma coagulation tests

Blood samples (1.2 ml) were collected from the rat femoral vein on the first and seventh days after modelling. Samples from each rat were placed in tubes with EDTA and sodium citrate (1:9), respectively. Samples in EDTA tubes were assessed using an automatic haematology analyser (PE-7060vet; Prokan Electronics INC., Shenzhen, China). Samples in sodium citrate tubes were assessed using an automatic coagulation analyser (H1201; Jiangsu Horner Medical Instrument Co., Jiangsu, China). Four coagulation indices including prothrombin time (PT), activated partial thromboplastin time (APTT), thrombin time (TT), and fibrinogen (FIB) were analysed to determine exogenous and endogenous coagulation as well as plasmin function.

### Haematoxylin and eosin (HE) staining

Rats were intraperitoneally injected with 1% sodium pentobarbital for deep anaesthesia. Cardiac perfusion was performed with 4% paraformaldehyde, following which the brains were carefully separated. Formaldehyde-fixed specimens were embedded in paraffin, cut into 4-μm sections, deparaffinised with xylene, and rehydrated in a graded series of alcohol. The slices were then observed and photographed under a microscope.

### Immunohistochemical analysis

Formaldehyde-fixed specimens were embedded in paraffin, cut into 4-μm sections, deparaffinised with xylene, and rehydrated in a graded series of alcohol. Antigen retrieval was performed by microwaving in a citric acid buffer. Sections were incubated with antibodies against erythropoietin (EPO) (1:800; Abcam, Cambridge, UK), vascular endothelial growth factor (VEGF)-A (1:400; Abcam), Claudin-5 (1:200; Abcam), and zonula occludens (ZO)-1 (1:100; Abcam) individually, washed, and then incubated with a secondary antibody for 1 h at room temperature. The same process was used for negative controls, except for antibody addition. A total of four sections from each rat were used for quantification, and protein expression was reflected by the optical density (OD) value, which was determined by two investigators blinded to the data using ImageJ (National Institutes of Health, Bethesda, USA).

### Nissl staining

Formaldehyde-fixed specimens were embedded in paraffin, cut into 4-μm sections, deparaffinised with xylene, and rehydrated in a graded series of alcohol. The sections were treated with Nissl staining solution (Aladdin Biochemical Technology Co, Shanghai, China) for 30 min. Neurons that were shrunken or contained vacuoles were considered damaged, whereas those with relatively large, full soma and round, large nuclei were considered normal [24]. Intact neuron counts were calculated using ImageJ by two investigators blinded to the experimental process.

### Enzyme-linked immunosorbent assay (ELISA)

Blood samples (1.2 ml) were collected from the rat femoral vein on the seventh day. Serum was separated by centrifugation and stored at − 80 °C until quantitative analysis of claudin-5 and ZO-1 using ELISA kits (Shanghai Westang Bio-tech Co., Shanghai, China). The measured OD values were then converted to a concentration value.

### TEM

After anaesthesia, the brain tissue (1 mm^3^, parasagittal brain tissue) was carefully separated and then fixed in 1.5% paraformaldehyde–3% glutaraldehyde for 24 h, followed by 1% osmic acid for 2 h. Conventional embedding and slicing for lead citrate and uranyl acetate staining were performed. The slices were then observed using TEM (JSM-7500F; Japan Electron Optics Laboratory Co., Japan). Vascular lumens and endothelial cells were observed at low magnification, while tight junctions (TJs) were observed at high magnification [25].

### Behavioural tests

The open-field test (OFT), elevated plus maze test, novel object recognition (NOR) test, and sucrose preference test were performed as previously described [26–28]. The OFT and elevated plus maze tests were used to detect anxiety-like behaviours [29, 30]. Each rat was placed at the centre of the open field (100 × 100 × 40-cm chamber; RWD Science Co., Shenzhen, China) for 5 min in a quiet room. A video-tracking system connected to a computer was used to record animal behaviour. The chamber was cleaned between experiments. For the elevated plus maze test, each rat was placed in the central area of the maze (50 × 10 × 30-cm close arm, 50-cm high; RWD).

The NOR test was used to assess learning and memory and was performed as previously described [31]. Twenty-four hours before testing, each rat was habituated for 5 min in a chamber. After the chamber had been cleaned, the rats were exposed to a set of two identical objects in the chamber for 10 min. Then, one of the objects was replaced with a novel object. The total interaction time was determined as the sum of the interaction times (familiar and novel objects). The discrimination index (%) was defined as the time spent exploring the novel object/total interaction time × 100. Rats with a total interaction time < 5 s were excluded from the analysis.

The sucrose preference test was used to assess behavioural anhedonia [28]. Briefly, two bottles of 1% sucrose solution were placed in each cage, and 24 h later, one bottle was replaced with drinking water for 24 h. After adaptation, rats were deprived of water and food for 24 h, followed by the sucrose preference test. In this test, rats housed in individual cages had free access to two bottles, one containing 200 ml of sucrose solution (1% w/v) and the other containing 200 ml of water. Levels of sucrose and water consumption (ml) were measured at 12 h and 24 h, and the sucrose preference was calculated as the volume of 1% sucrose solution consumed, expressed as a percentage of the total liquid intake. All behavioural tests were performed on the seventh day after modelling.

### Statistical analysis

Data are presented as the mean ± standard deviation (SD). Statistical analyses were performed using SPSS 18.0 (SPSS, Inc., Chicago, IL, USA) and R software (version 4.1.1). The “ggplot2” R package was used to perform principal component analysis (PCA) and generate a hierarchical clustering heatmap showing the coincidence of indicators between animal models and clinical patients. Data distributions were assessed using the Kolmogorov–Smirnov nonparametric test of equality. Differences between two groups were assessed using Student’s t-test (normally distributed parameters) or Mann–Whitney U-tests (non-normally distributed parameters). Statistical significance was set at P < 0.05.

## Results

### Patient characteristics

A total of 48 patients with CVST (22 men and 26 women, mean age: 39.90 ± 14.66 years) were included in this study (Additional file 1: Table S1). CVST was acute in 18 patients (37.50%) and subacute in 30 patients (62.50%). Demographic data, past medical history, clinical characteristics, and laboratory test results for these patients and 48 healthy controls are shown in Additional file 1: Table S1.

When compared with the control group, the CVST group exhibited increased white blood cell (WBC) count (P < 0.001), neutrophil percentage (NEU%) (P < 0.001), ADC value (P < 0.001), and ICP (P < 0.001), as well as shortened APTT (P < 0.001) and TT (P < 0.01) (Additional file 1: Table S1). Follow-up information indicated that all 34 patients with available data experienced good outcomes, although four and three cases of anxiety and depression were observed (with both occurring in two cases), respectively.

### SSS occlusion monitoring

LSCI can generate cerebral blood flow (CBF) maps for monitoring changes in blood flow on the brain surface. The posterior segment of the SSS was not immediately occluded following rubber insertion (Fig. 1d). On the first day, the rubber expanded to fill the posterior part of the SSS, and the blood flow in the SSS and bridging veins nearly disappeared (Fig. 1d). Consistently, the MRA reconstruction maps on both the first and seventh days showed complete disappearance of the SSS (Additional file 1: Fig. S2). Near-complete disappearance of the transverse sinuses was also observed, indicative of extensive thrombosis after SSS occlusion (Additional file 1: Fig. S2). These observations suggested that CVST models induced by rubber insertion can provide stable and reproducible SSS occlusion.

### Vascular morphology was similar in animal models and patients with CVST

**Fig. 2:**
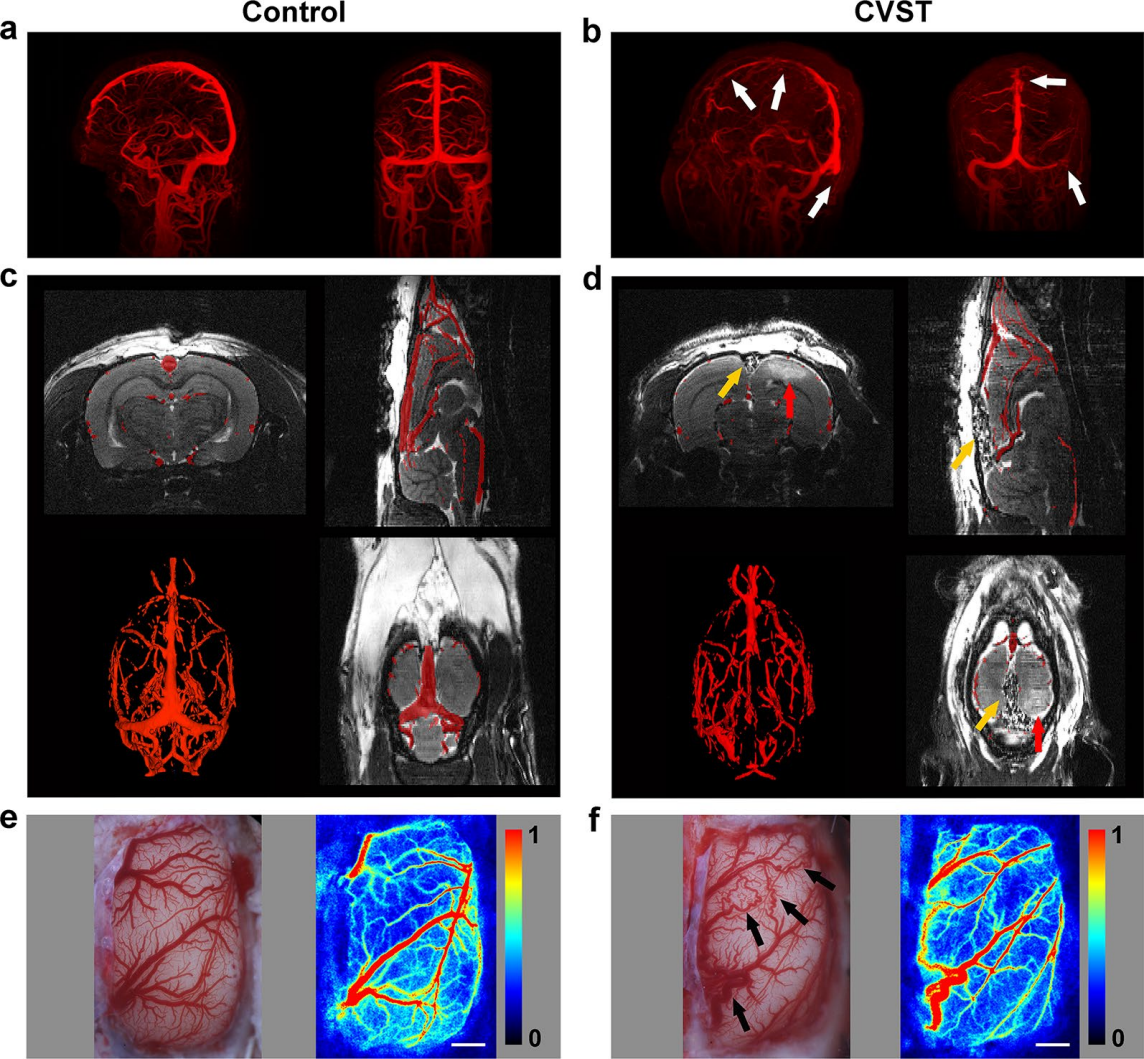
Vascular morphology in patients with CVST and animal models. **a** MRV reconstruction in the healthy control group showing no abnormalities in vascular anatomy. **b** MRV reconstruction in patients with CVST showing filling defects in the SSS, right transverse sinus and sigmoid sinus (white arrows). **c**, **d** T2WI and MRA maps. **c** No abnormality of cerebral vascular anatomy in the sham group. **d** In the model group, the SSS is filled with swellable rubber, without blood flow (yellow arrows), and a high-signal area can be observed near the SSS (red arrows). **e**, **f** Microscope photographs and CBF maps obtained via LSCI. **e** In the sham group, all veins are normal. **f** In the model group, the parasagittal veins are distorted and tortuous, and anastomotic pathways have formed between the distorted veins (black arrows). Red to blue color bars on the right side represent blood flow from high to low. Scale bar = 1 mm. CVST: cerebral venous sinus thrombosis; MRV: magnetic resonance venography; T2WI: T2-weighted imaging; MRA: magnetic resonance angiography; SSS: superior sagittal sinus; CBF: cerebral blood flow; LSCI: laser-speckle contrast imaging

In patients with CVST, venous morphology was characterised by distorted surrounding veins and intricate vascular pathways around the occluded venous sinus (Fig. 2b). To identify changes in vascular morphology in animal models, we performed MRA scanning on the first and seventh days after modelling, observing the same pattern of venous morphology as in humans. The course and shape of veins around the SSS were obviously distorted (Fig. 2d). LSCI results further validated these findings. On the seventh day, veins adjacent to the SSS were tortuous and dilated, forming new anastomotic pathways, with unobstructed blood flow in the anastomotic branches (Fig. 2f).

### Lesion features on imaging were similar in animal models and patients with CVST

**Fig. 3:**
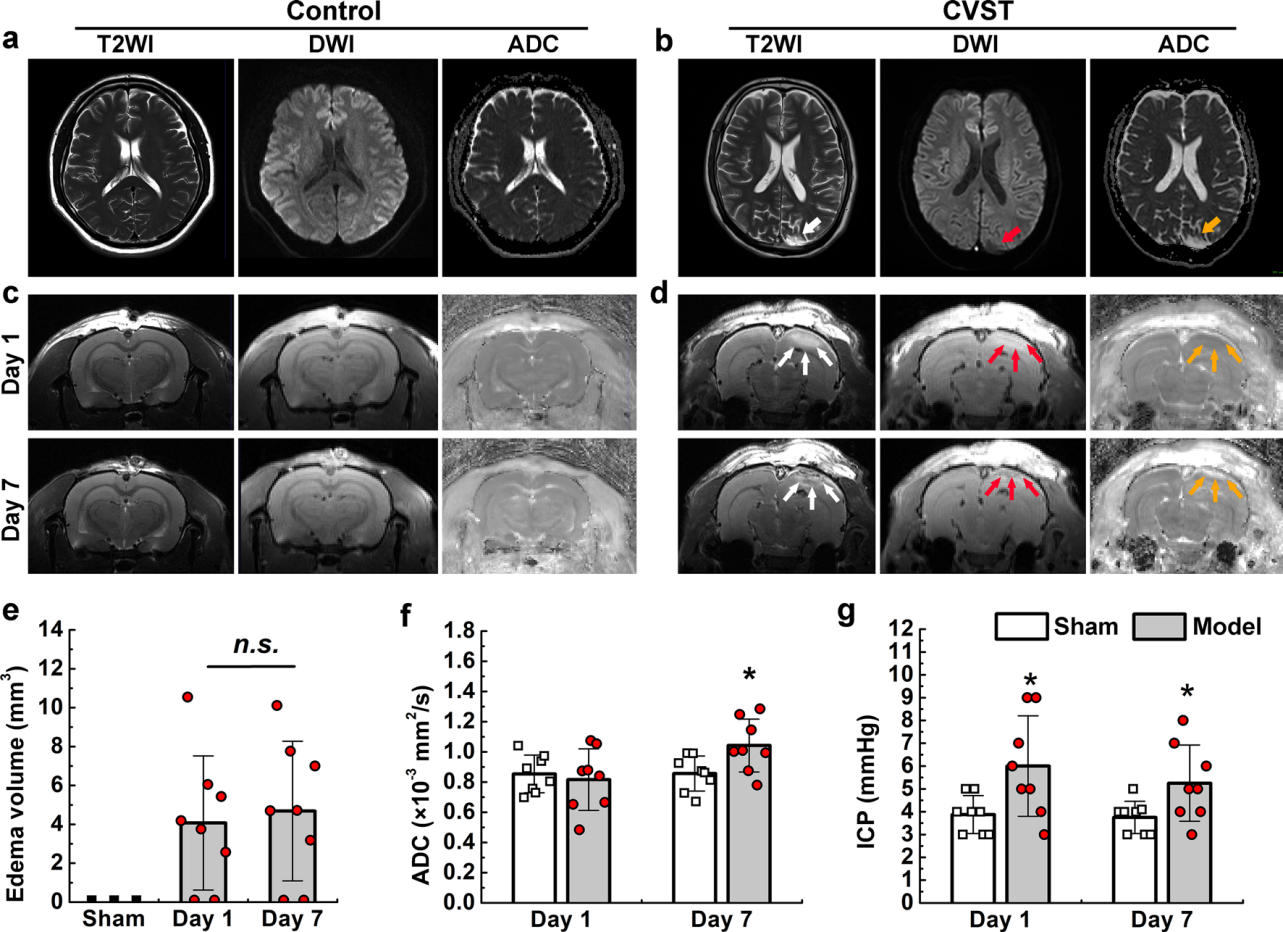
Imaging features of lesions in patients with CVST and animal models. **a** The T2WI, DWI, and ADC maps are normal in the healthy control. **b** An area with slightly high signal can be observed in the occipital lobe on T2WI (white arrow), presenting as a low signal area on DWI (red arrow) and a high signal area on ADC (yellow arrow), in patients with CVST. **c** Images from the first and seventh days after modelling showing no obvious abnormalities in the sham group. **d** Image from the model group showing a high-signal area on the right side near the SSS on T2WI (white arrows) and DWI (red arrows) maps and a low signal area on ADC map (yellow arrows) on the first day. The image from the seventh day shows some scattered low-density fields on T2WI (white arrows) and DWI (red arrows) maps and a high signal area on ADC map (yellow arrows) at the original oedemic area, indicating the possibility of venous haemorrhage. **e** Brain oedema volume quantified based on T2WI in animal models. **f** ADC quantification for model rats. Values are expressed as mean standard deviation. n.s., P > 0.05, *P < 0.05. CVST: cerebral venous sinus thrombosis; T2WI: T2-weighted imaging; SSS: superior sagittal sinus; DWI: diffusion-weighted imaging; ADC: apparent diffusion coefficient

We also performed T2WI and DWI scanning in animal models on the first and seventh days after modelling, which revealed brain oedema adjacent to the occluded SSS (Fig. 3d). The volume of brain oedema measured based on T2WI was 4.07 ± 3.45 mm^3^ on the first day and 4.68 ± 3.59 mm^3^ on the seventh day (Fig. 3e). ADC values were also used to evaluate brain oedema. These values were lower in the model group than in the sham group on the first day (0.82 ± 0.20 mm^2^/s vs. 0.85 ± 0.13 mm^2^/s, P > 0.05) but higher on the seventh day (1.04 ± 0.18 mm^2^/s vs. 0.86 ± 0.12 mm^2^/s, P < 0.05) (Fig. 3f). Patients with CVST also exhibited significantly higher ADC values (0.99 ± 0.24 mm^2^/s) than the control group (0.76 ± 0.05 mm^2^/s; P < 0.001) (Additional file 1: Table S2). The cluster analysis heatmap further verified that ADC values were higher in the CVST group than in the control group (Fig. 5b). In animal models, ICP values increased significantly on both the first and seventh days when compared with values in the sham group (P < 0.05) (Fig. 3g). Consistently, intracranial hypertension was also observed in human patients (Additional file 1: Table S2).

### Haematological changes were similar in animal models and patients with CVST

**Fig. 4:**
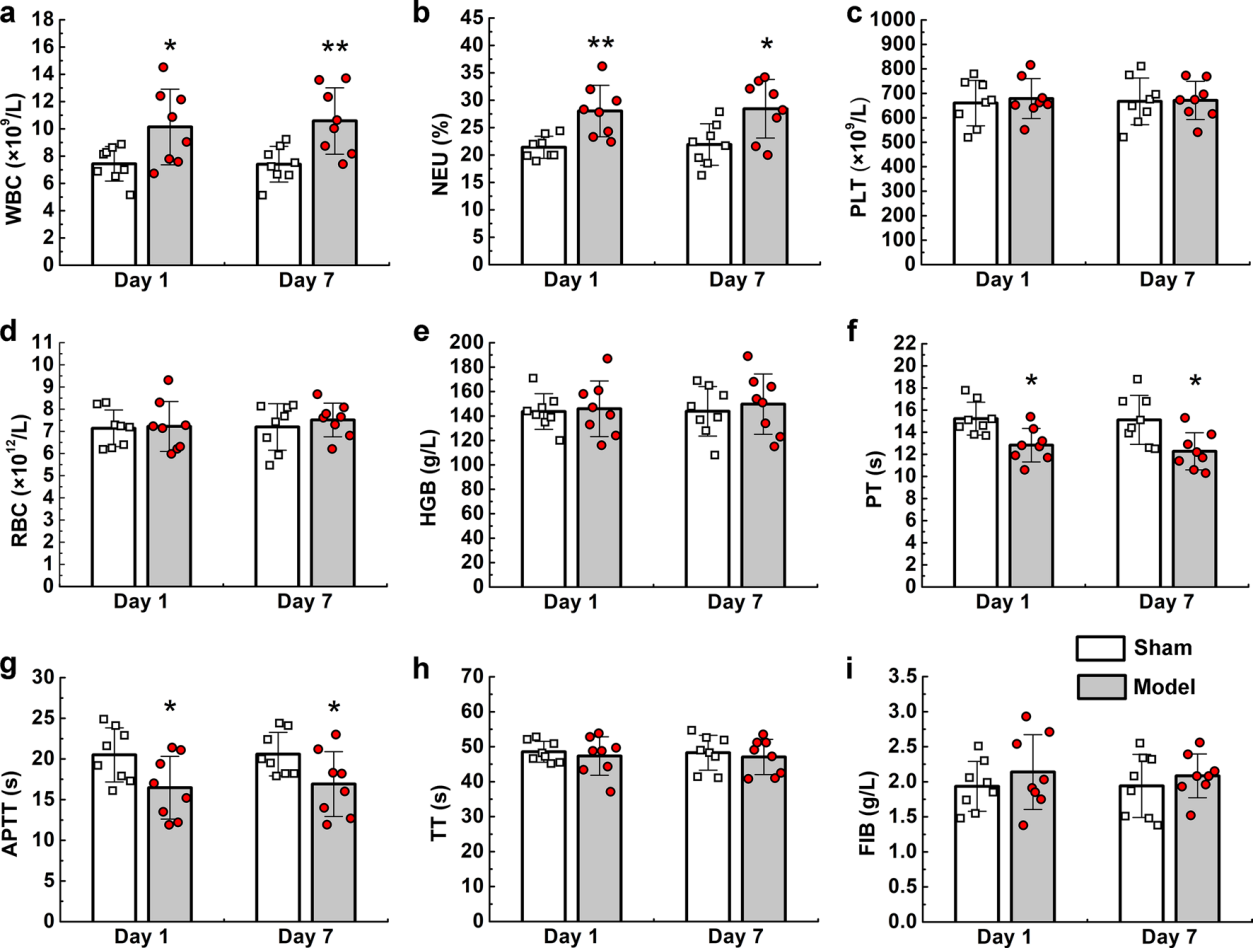
Routine blood and plasma coagulation tests in animal models. Routine blood test (**a**–**e**) and plasma coagulation (**f**–**i**) results on the first and seventh days in animal models. Values are expressed as mean standard deviation. *P < 0.05, **P < 0.01. WBC: white blood cell; NEU: neutrophil; PLT: platelet; RBC: red blood cell; HGB: hemoglobin; PT: prothrombin time; APTT: activated partial thromboplastin time; TT: Thrombin time; FIB: fibrinogen

Routine blood and coagulation tests were performed to examine CVST-related changes in blood components. WBC and NEU% were significantly increased in the model group when compared with values in the control group on the first and seventh days (Fig. 4a, b), although there were no obvious changes in red blood cell or platelet counts. Coagulation analyses indicated that PT and APTT were significantly shortened in the model group (Fig. 4f, g), while there were no obvious changes in TT or FIB.

**Fig. 5:**
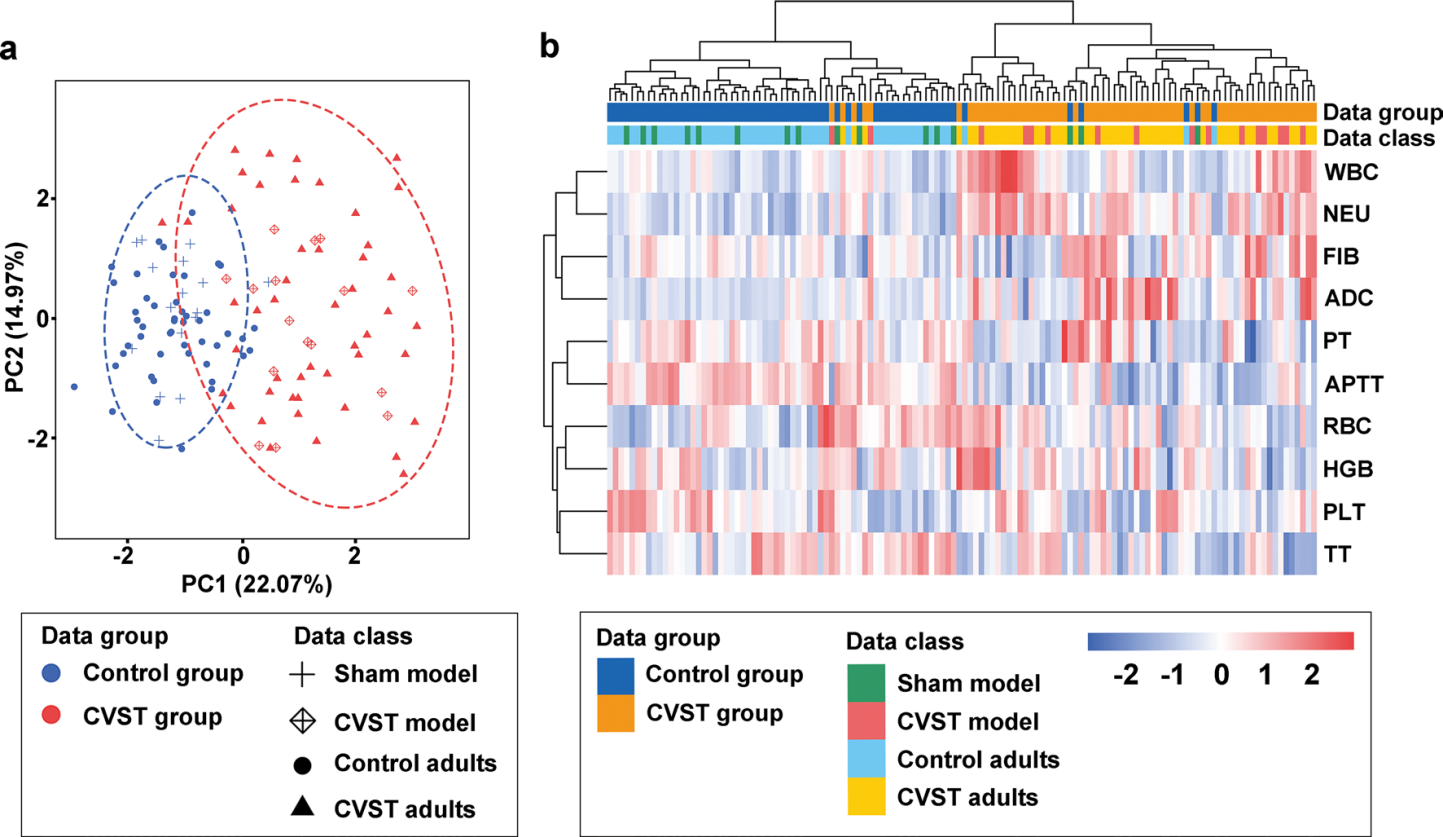
Analysis of similarity between human patients and animal models. **a** PCA and **b** a hierarchical clustering heatmap revealed that data for clinical patients and animal models were highly clustered in the control or CVST groups. CVST: cerebral venous sinus thrombosis; PCA: Principal component analysis

PCA was performed to examine the similarity in variable changes between clinical patients and animal models (Fig. 5a). This method enables reduction of a highly multidimensional dataset into two dimensions, or principal components, allowing for unbiased comparisons and visualisation of clinical and animal data. PCA indicated that clinical and animal variables presented a high degree of clustering in the control group or CVST group (Fig. 5a). We next performed hierarchical clustering analysis, and, consistent with PCA, the dendrogram showed a coincidence of animal and clinical distributions (Fig. 5b). The heatmap revealed significant differences in WBC, NEU%, and APTT between the control and CVST groups (Fig. 5b).

### CVST-related damage to the brain parenchyma in animal models

**Fig. 6:**
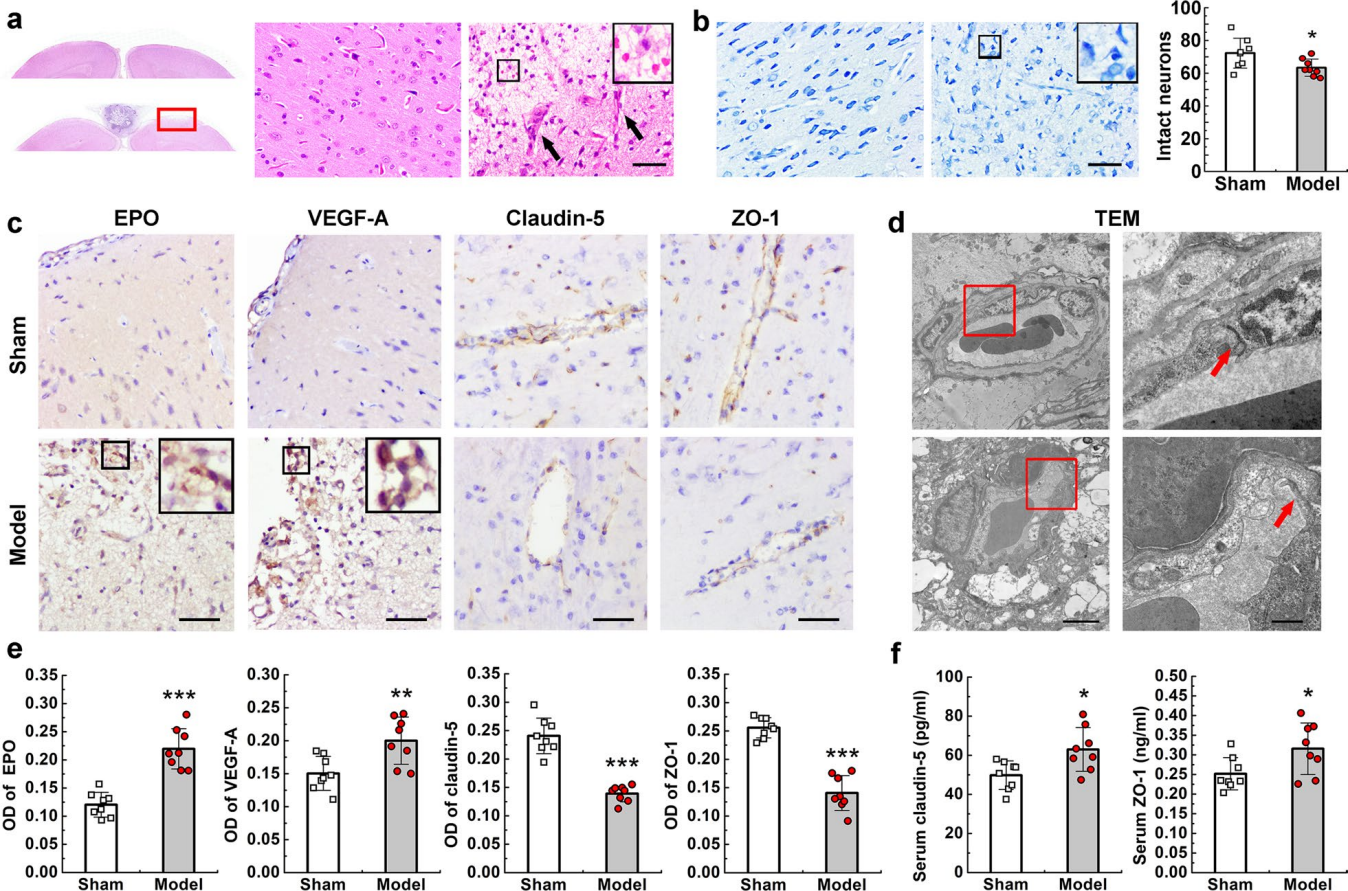
Damage to the brain parenchyma characterised by vasogenic oedema in animal models. **a** HE staining showing filling of the SSS with the expanded rubber implant. Microscopic image showing erythrocyte scattering and interstitial enlargement around the oedemic area, with increases in capillary-like structures (black arrows). **b** Nissl staining showing an obvious decrease in the number of normal neurons and abnormal arrangement of cells. **c** Immunohistochemical sections showing increased expression of EPO and VEGF-A and decreased expression of claudin-5 and ZO-1 around the oedemic area. **d** TEM results showing the vascular lumen and endothelial cells under low power (× 15,000) and TJs located between endothelial cells under higher power (×50,000). The intact TJ appears as a black-dense band in the sham group (red arrow). This band is almost disappeared in the model group (red arrow), which exhibited interstitial enlargement around the vascular lumen. **e** Quantification of immunohistochemical analysis. **f** Quantification of claudin-5 and ZO-1 in the serum. Scar bars = 100 μm in (**a**–**c**), Scar bars = 2 μm on the lower power lens maps and 500 nm on the higher power lens maps in **d**. Values are expressed as mean standard deviation. *P < 0.05, **P < 0.01, ***P < 0.001. SSS: superior sagittal sinus; EPO: erythropoietin; VEGF-A: vascular endothelial growth factor A; ZO-1: zonula occludens 1; TEM: transmission electron microscopy; TJ: tight junction

Histological analysis of HE-stained specimens revealed filling of the expandable rubber implant in the model group. As observed on imaging, oedema was present near the occluded SSS (Fig. 6a). Furthermore, microhaemorrhages were observed in some animals (3/8) around the oedemic area. To further evaluate the degree of neuronal injury, numbers of intact neurons were quantified using Nissl staining [24]. In the model group, several abnormal neurons featuring pyknotic nuclei and shrunken cytoplasm were observed in the oedemic area, while few such neurons were observed in the sham group (Fig. 6b). Quantitative analysis revealed a statistically significant difference between the two groups (P < 0.05).

We then analysed the presence of vasogenic oedema in each group. Previous studies have reported that EPO and VEGF-A are important proteins promoting angiogenesis, but that they may exacerbate vasogenic oedema [32, 33]. Immunohistochemical sections revealed that EPO and VEGF-A expression was significantly increased in the oedemic area (Fig. 6c, e). TJ integrity was also evaluated to assess the function of the blood–brain barrier (BBB) [34, 35]. Immunohistochemical sections revealed significantly decreased expression of claudin-5 and ZO-1 in the model group (P < 0.001) (Fig. 6c, e), while serology revealed significantly increased serum concentrations of claudin-5 and ZO-1 in the model group (P < 0.05) (Fig. 6f). TEM further confirmed the splitting of TJs in the model group, as well as interstitial enlargement around the vessel lumen (Fig. 6d). These pathological consequences support the predominance of vasogenic oedema.

### Neurological dysfunction in CVST models

**Fig. 7:**
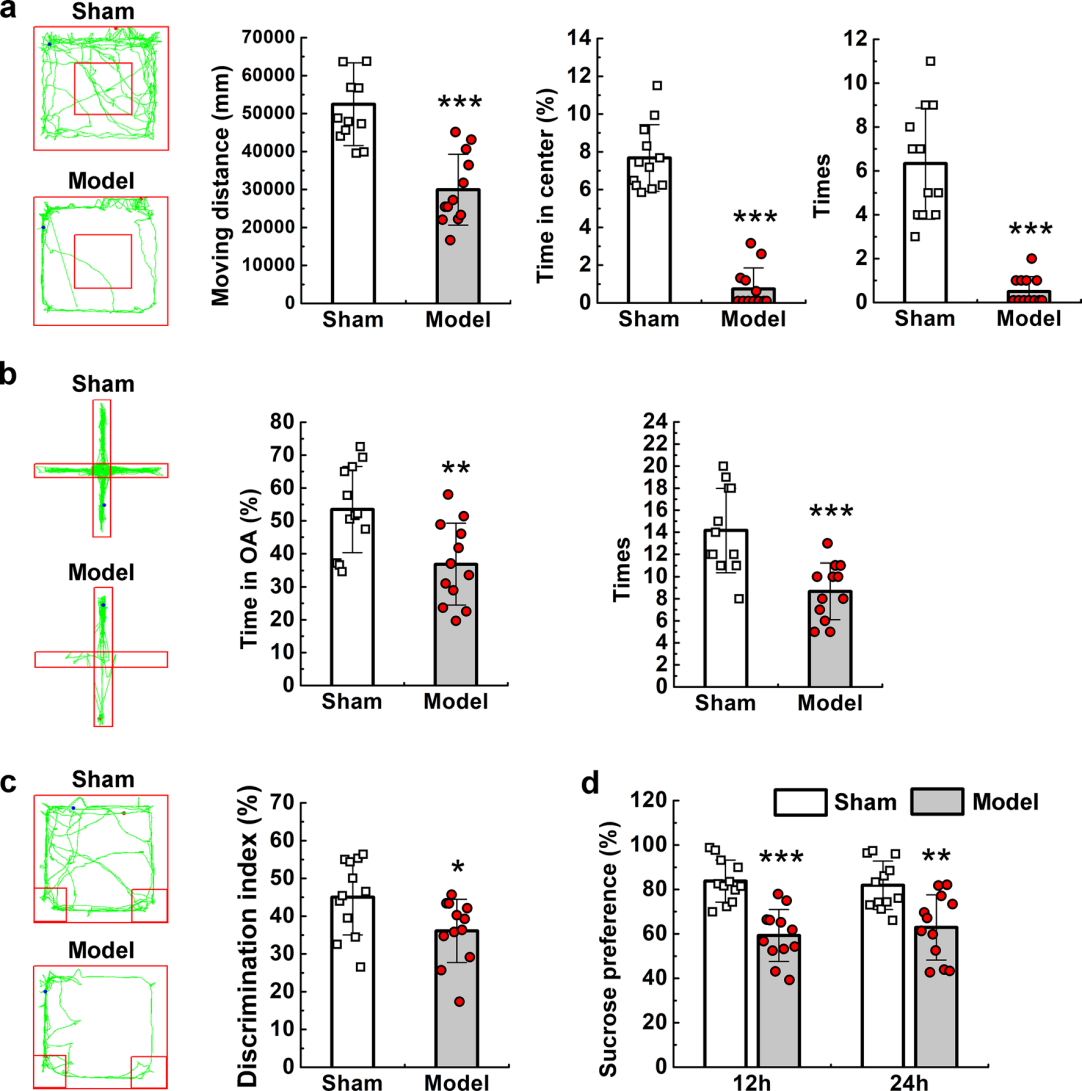
Neurological dysfunction in animal models. **a** OFT activity traces over 5 min showing significant differences in distance travelled, time spent in the central area, and number of central crossings between the sham and model groups. **b** Elevated plus maze test activity traces over 5 min showing significant differences in time spent in the open arms and the number of entries through intersection of open and closed arms between the sham and model groups. **c** NOR test activity traces and the discrimination index. **d** Sucrose preference test results at 12 h and 24 h. Values are expressed as mean standard deviation. *P < 0.05, **P < 0.01, ***P < 0.001. OFT: open-field test; OA, open arms; NOR: novel object recognition

To determine whether parenchymal injury was sufficient to impair neurological function, we conducted a variety of behavioural tests. In the OFT and elevated plus maze tests, the time spent in the central area (P < 0.001), time spent in open arms (OAs) (P < 0.01), and number of central crossings (P < 0.001) were significantly decreased in model rats when compared with those in sham rats (Fig. 7a, b). In the sucrose preference test, decreased sucrose consumption was observed in model rats at both 12 h (P < 0.001) and 24 h (P < 0.01) (Fig. 7d). These results suggest the presence of anxiety-like and depression-like behaviours in model rats. In the NOR test, the model group had a lower discrimination index (P < 0.05) than the sham group (Fig. 7c), indicating the possibility of decreased learning and memory ability. No rats died during this study.

## Discussion

**Fig. 8:**
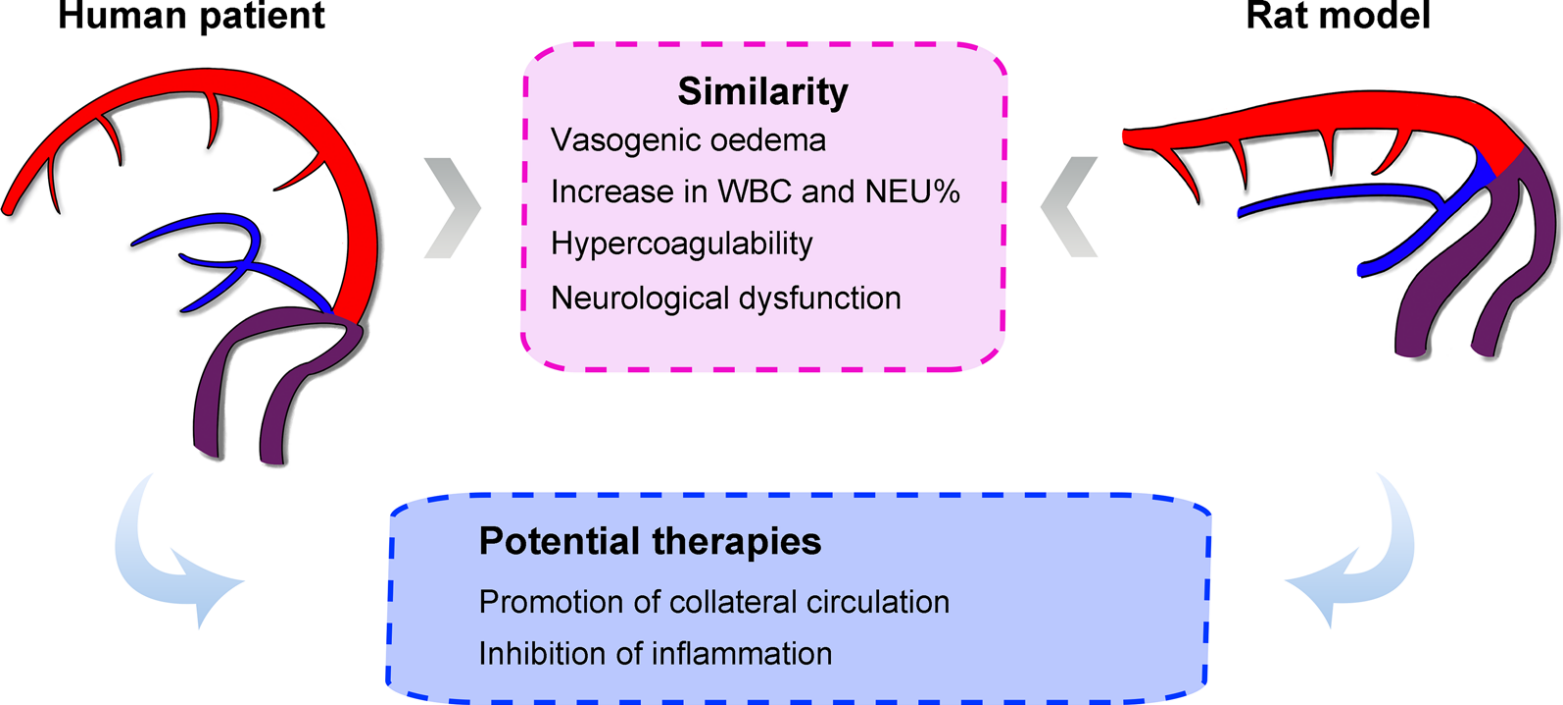
Verification of similarity between clinical patients and animal models

In this study, we successfully established a novel CVST rat model by inserting a water-swellable rubber implant into the SSS and verified the stability of SSS occlusion using 9.4-T small-animal MRI and LSCI. Imaging results indicated a possibility of vasogenic oedema in animal models, while haematological analysis revealed an increase in WBC and NEU% accompanied by a decrease in APTT and PT. These findings were mostly consistent with the retrospective clinical data for human patients. Further PCA and cluster analysis revealed a high degree of clustering for the clinical and animal datasets, validating the reproducibility of the model and its similarity to clinical cases. In addition, pathological and serological tests in animal models indicated that vasogenic oedema was the dominant type, as evidenced by increased expression of angiogenic proteins and BBB disruption in the oedemic area (Fig. 8).

Given that the pathophysiology of CVST remains to be fully clarified, an animal model with a high degree of clinical similarity is required. While several animal models of CVST have been constructed over the past few decades, most of these models involved SSS surgery, as the SSS is the most susceptible vessel in human CVST [1] and is easy to expose, reducing the trauma associated with modelling. The first model established was the SSS ligation model, which is simple to generate but requires an invasive operation that can cause irreversible damage to the parasagittal brain tissue and collateral circulation, reducing the reliability of the pathology [8, 9]. Subsequently, Ungersböck et al. and Frerichs et al. injected procoagulant into the occluded SSS segment to increase the stability of thrombosis on the basis of the SSS ligation model [11, 12]. Compared with ligation alone, thrombi induced by coagulants were more extensive and persistent. However, because of the lack of valves in the cerebral venous system, the coagulants can enter the systemic circulation via collateral circulation, leading to thrombosis in multiple extracranial tissues. To avoid mechanical injury, Röttger et al. applied a piece of filter paper soaked with 40% ferric chloride solution to the surface of the SSS. However, the model exhibited a high spontaneous recanalisation rate, making it unsuitable for long-term studies [36]. Moreover, the toxicity of ferric chloride itself can lead to corrosion of the cerebral cortex, resulting in false-positive identification of damage to the brain parenchyma. Although the pathophysiological process of thrombosis induced by the photochemical method was in greater accordance with the clinical data, it can also lead to arterial thrombosis in the irradiated area [37].

In a previous study, we established a CVST model by inserting a self-made thread plug into the rat SSS. However, with gradual centripetal widening of the SSS, it was difficult to completely occlude the posterior segment of the SSS [13]. No obvious parenchymal damage was observed after SSS occlusion. This defect was modified in the present study by using swellable rubber, allowing for proper expansion in the blood. This expansive property results from the polyurethane-based water-expanding polymer resin, which can expand to approximately 250% of its volume when in contact with water [38, 39]. Both MRA and LSCI showed complete occlusion of the middle and posterior segments of the SSS, while histology further confirmed that the material fully filled the lumen of the SSS.

Cerebral ischaemia can induce brain oedema, which can be divided into cytotoxic oedema and vasogenic oedema. These two types can be distinguished based on ADC values extracted from DWI, which present as decreases and increases in ADC values respectively [40]. Previous clinical studies have reported that vasogenic oedema develops earlier and is predominant in patients with CVST, although cytotoxic oedema is also involved in CVST-related pathological changes [22, 41]. Our clinical data support the notion that ADC values are increased in patients with CVST. In animal models, ADC values decreased slightly on the first day, although they were increased on the seventh day. Srivastava et al. used ferric chloride to induce CVST in rats, reporting a more severe decrease in ADC and subsequent recovery after modelling [42]. This may be attributed to the prompt occlusion of SSS induced by ferric chloride, leading to early development of cytotoxic oedema. Our model partially mitigates that risk of cytotoxic oedema due to the delay in material swelling. Recovery or elevation of ADC may be related to the compensatory effects of collateral circulation. In contrast to cerebral artery thrombosis, which almost always causes irreversible damage, collateral circulation can still maintain a lower flow rate of blood perfusion through affected brain lesions after venous thrombosis [12]. Moreover, hypoxic conditions may further enrich collateral circulation. In the present study, proliferation of capillary-like structures adjacent to the occluded SSS was observed on the seventh day after modelling. Elevated expression of the angiogenic proteins EPO and VEGF-A further suggested compensatory proliferation of collateral circulation around the oedemic area. However, studies have highlighting the two-pronged effects of angiogenic proteins, which not only induce angiogenesis but can lead to BBB damage [32, 33]. In the present study, both decreased TJ protein expression and TJ disconnection in oedemic areas further confirmed the disruption of the BBB in animal models. These pathological changes further illustrate that CVST is associated with vasogenic oedema.

Our clinical analysis indicated that patients with CVST exhibited increased ICP when compared with healthy controls. Similarly, intracranial hypertension was also observed in animal models. The Monroe–Kellie theory states that the total volumes of cerebral blood, cerebrospinal fluid, and brain tissue are relatively constant because the skull cavity is an airtight rigid structure [43]. Cerebrospinal fluid drainage is severely affected following the obstruction of venous return, and accumulation of blood and cerebrospinal fluid may lead to increased ICP [7, 43]. However, ICP was slightly decreased on the seventh day when compared with the first day in animal models, which may reflect the compensatory effect of collateral circulation. Histological findings revealed capillary proliferation and increased expression of angiogenic proteins around oedemic areas, both of which reasonably support this speculation. In fact, collateral circulation is demonstrated to play a beneficial role in the pathophysiology of stroke, brain injury, and cerebral diseases [32, 44, 45]. Similarly, collateral circulation may represent a therapeutic target in CVST.

In this study, WBC and NEU% were increased in both patients with CVST and animal models. Several previous studies have reported a relationship between inflammation and venous thrombosis. [46–48]. One in vivo noted that neutrophils contribute to the initiation of thrombosis [49]. Similarly, Darbousset et al. found that binding of neutrophils to the vessel wall is a major source of tissue factor in the early stage of thrombosis [50]. Therefore, inhibition of neutrophil-induced thrombosis may have therapeutic value for CVST. Coagulation tests revealed decreased APTT in the CVST group, while PT and APTT were shortened in the animal model, both suggesting hypercoagulability after CVST. The difference may have been caused by the effect of the embolus on extrinsic coagulation pathways in models.

The study had several limitations. Since SSS occlusion in the animal model is caused by material expansion, spontaneous recanalisation does not occur during the pathophysiological process. However, research has indicated that patient outcomes are not dependent on recanalisation or persistent occlusion of the venous sinus [51]. Therefore, this defect may not inhibit further exploration of pathophysiology, such as the mechanism of collateral circulation. Furthermore, few studies have reported neurological function and mental status in the context of CVST. Bourrienne et al. established a CVST animal model and observed dysfunction of neurological sensory-motor functions [10]. Similarly, admission scores (GCS, NIHSS, and mRS) in the present study suggest that patients with CVST exhibit neurological deficits, while behavioural tests suggested anxiety and depression-like changes in model rats. As four and three cases of anxiety and depression were noted in the 34 patients with follow-up data, respectively, further research regarding neurological prognosis and mental status after CVST is required.

## Conclusion

In the present study, we successfully established a novel rat model of CVST. Cluster analysis of haematological and imaging data indicated high similarity of clinical features in human patients and animal models. Histological results further validated the characteristic pathological changes related to venous thrombosis in the model. This model provides reliable support for the study of CVST pathophysiology and potential therapies.

## Supplementary Information

**Additional file 1.** Additional figures and tables.

## Data Availability

The dataset supporting the conclusions of this article is included within the article.

## Abbreviations

ADC: Apparent diffusion coefficient
APTT: Activated partial thromboplastin time
BAI: Beck Anxiety Inventory
BDI: Beck Depression Inventory
CVST: Cerebral venous sinus thrombosis
DWI: Diffusion-weighted imaging
ELISA: Enzyme-linked immunosorbent assay
EPO: Erythropoietin
FIB: Fibrinogen
FOV: Field-of-view
GCS: Glasgow Coma Scale
HAMD: Hamilton Depression Scale
HE: Haematoxylin and eosin
ICP: Intracranial pressure
LSCI: Laser-speckle contrast imaging
MRA: Magnetic resonance angiography
mRS: Modified Rankin scale
NEX: Number of excitations
NIHSS: National Institutes of Health Stroke Scale
NOR: Novel object recognition
OA: Open arm
OD: Optical density
OFT: Open-field test
PCA: Principal component analysis
PT: Prothrombin time
ROI: Region of interest
SD: Standard deviation
SSS: Superior sagittal sinus
T2WI: T2-weighted imaging
TE: Echo time
TJ: Tight junction
TEM: Transmission electron microscopy
TR: Repetition time
TT: Thrombin time
VEGF: Vascular endothelial growth factor
ZO-1: Zonula occludens
