# Supplementary material for: A novel rat model for cerebral venous sinus thrombosis: verification of similarity to human disease via clinical analysis and experimental validation
Source: J Transl Med. 2022 Apr 11;20:174. doi: 10.1186/s12967-022-03374-y (PMC8996223; doi:10.1186/s12967-022-03374-y)
Supplement: Supplementary file 1 — Additional file 1. Additional figures and tables. [file 12967_2022_3374_MOESM1_ESM.docx]

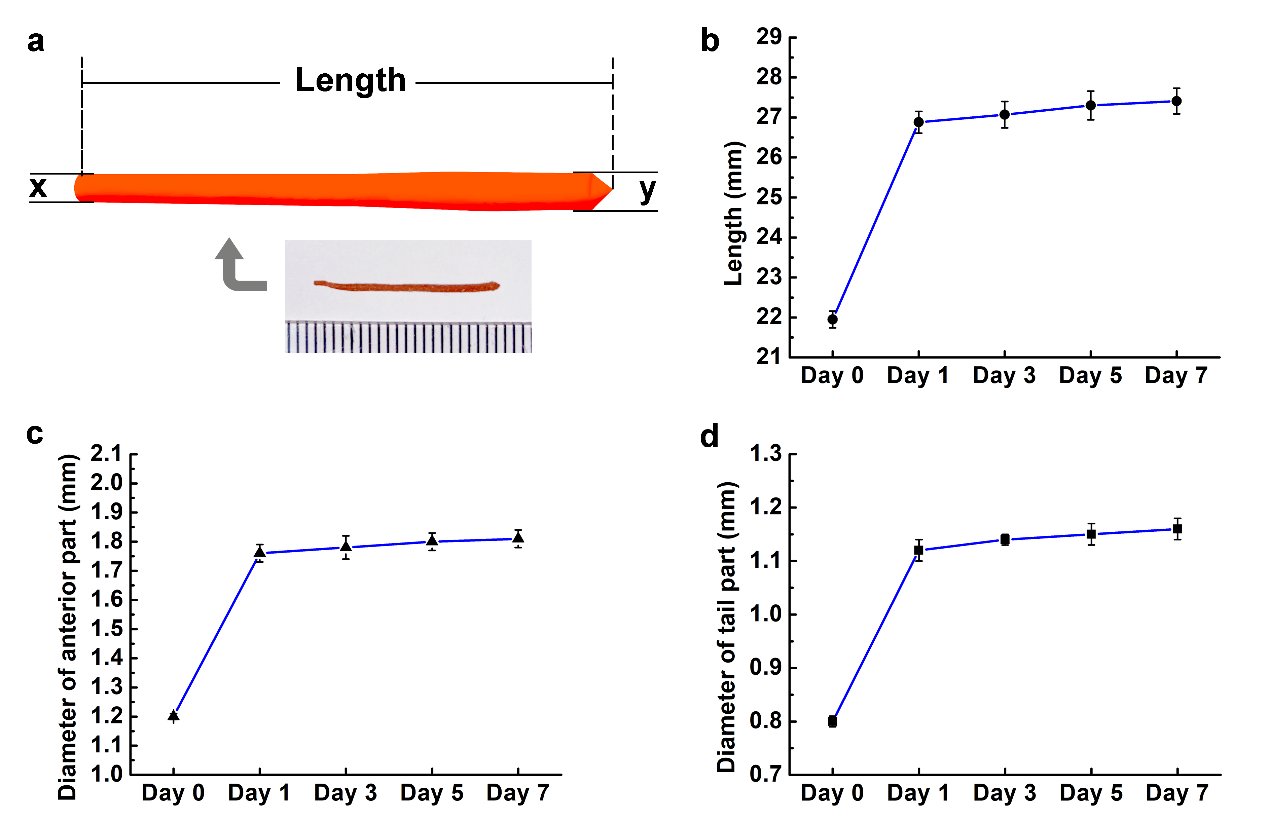


**Fig. S1 Expansion test of water swellable rubber in vitro.** (a) Specification of the water swellable rubber: (x) tail part, (y) anterior part. (b-d) Rat venous blood was mixed with anticoagulant and the rubber was immersed in the blood to observe rubber expansion over time. Day 0 means initial specification. The specification data of the rubber was basically stable from the first day; there was no significant difference between any pairs among day 1, day 3, day 5, and day 7.


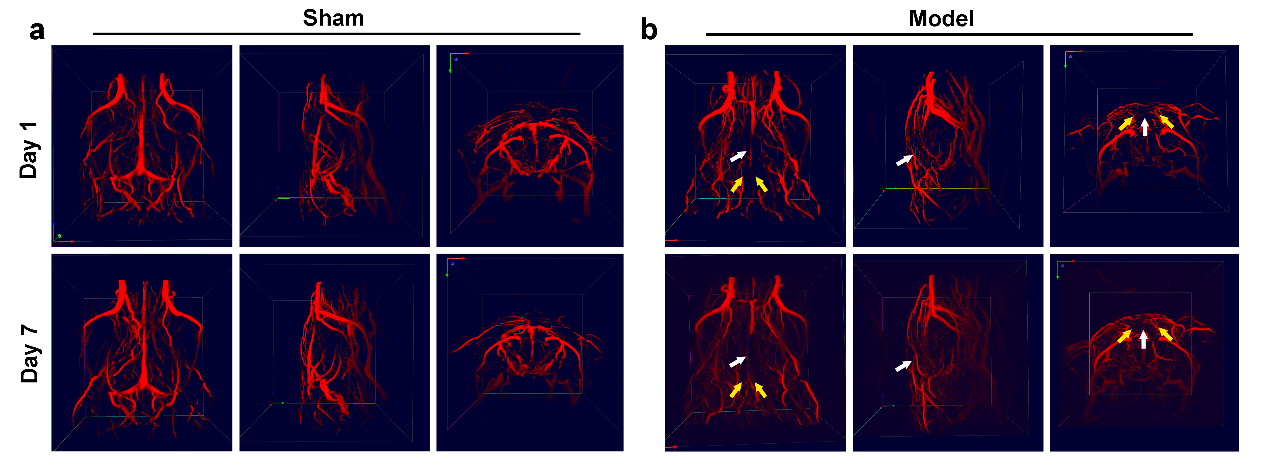


**Fig. S2 Three-dimensional MRA reconstruction.** (a) MRA clearly showing normal cerebrovascular anatomy of rats in the sham group. (B) In the model group, MRA on both the first and seventh days showed complete disappearance of the SSS (white arrows). Near-complete disappearance of the transverse sinuses was also observed (yellow arrows). MRA, magnetic resonance angiography, SSS, superior sagittal sinus.

**Table S1** Demographic features and clinical characteristics of CVST patients.

| **Characteristics** | **Total (n=48)** | **Acute (n=18)** | **Subacute (n=30)** |
| --- | --- | --- | --- |
| **Demographics** |  |  |  |
| Age, yrs | 39.90 ± 14.66 | 40.50 ± 3.91 | 39.53 ± 2.49 |
| Gender, female | 26 (54.17%) | 9 (50.00%) | 17 (56.67%) |
| **Medical history** |  |  |  |
| Smoking history | 11 (22.92%) | 4 (22.22%) | 8 (26.67%) |
| Hypertension | 9 (18.75%) | 3 (16.67%) | 6 (20.00%) |
| Diabetes mellitus | 5 (10.42%) | 2 (11.11%) | 3 (10.00%) |
| Dyslipidemia | 15 (31.25%) | 5 (27.78%) | 10 (33.33%) |
| Abnormal liver function | 10 (20.83%) | 4 (22.22%) | 6 (20.00%) |
| **Admission clinical scoring** | |  |  |
| mRS | 1 (1-3) | 2 (1-4) | 1 (1-2) |
| NIHSS | 1.5 (0-8) | 6.5 (0-12) | 0.5 (0-4) |
| GCS | 13.77 ± 2.43 | 12.56 ± 3.20 | 14.50 ± 1.43 |
| **Venous sinus involvement** | | |  |
| Sagittal sinus thrombosis | 33 (68.75%) | 12 (66.67%) | 21 (70.00%) |
| Transvers sinus thrombosis | 26 (54.17%) | 11 (61.11%) | 15 (50.00%) |
| Sigmoid sinus thrombosis | 23 (47.92%) | 9 (50.00%) | 14 (46.67%) |
| Cortical vein thrombosis | 24 (50.00%) | 10 (55.56%) | 14 (46.67%) |
| Intracranial deep vein thrombosis | 9 (18.75%) | 4 (22.22%) | 5 (16.67%) |
| cerebral ischemia | 20 (41.67%) | 8 (44.44%) | 12 (40.00%) |
| cerebral hemorrhage | 17 (35.42%) | 7 (38.89%) | 10 (33.33%) |
| ADC, ×10^-3^ mm^2^/s | 0.99 ± 0.25 | 0.95 ± 0.23 | 1.02 ± 0.25 |
| **Laboratory** |  |  |  |
| WBC, ×10^9^/L | 8.46 ± 2.47 | 8.93 ± 2.06 | 8.18 ± 2.68 |
| NEU, % | 72.48 ± 8.87 | 74.26 ± 7.04 | 71.42 ± 9.76 |
| RBC, ×10^12^/L | 4.54 ± 0.47 | 4.52 ± 0.41 | 4.56 ± 0.51 |
| HGB, g/L | 131.83 ± 16.15 | 133.56 ± 16.25 | 130.80 ± 16.28 |
| PLT, ×10^9^/L | 211.60 ± 64.73 | 196.39 ± 54.48 | 220.73 ± 69.42 |
| PT, s | 10.90 ± 1.14 | 10.93 ± 1.19 | 10.89 ± 1.13 |
| APTT, s | 31.33 ± 4.12 | 31.54 ± 4.36 | 31.20 ± 4.03 |
| TT, s | 15.99 ± 1.64 | 16.18 ± 1.85 | 15.87 ± 1.52 |
| FIB, g/L | 3.08 ± 1.06 | 3.07 ± 1.22 | 3.08 ± 0.98 |
| **Monitoring** |  |  |  |
| ADC, ×10^-3^ mm^2^/s | 0.99 ± 0.24 | 0.95 ± 0.23 | 1.02 ± 0.25 |
| ICP^a^, mmH_2_O | 218.33 ± 62.30 | 217.78 ± 75.17 | 218.67 ± 54.57 |

a: Data of ICP were derived from cerebrospinal fluid pressure recorded by lumbar puncture.

ADC: apparent diffusion coefficient; APTT: activated partial thromboplastin time; CVST: cerebral venous sinus thrombosis; FIB: fibrinogen; GCS: Glasgow Coma Scale; HGB: hemoglobin; ICP: intracranial pressure; mRS: modified Rankin Scale; NEU: neutrophil; NIHSS: National Institute of Health Stroke Scale; PLT: platelet; PT: prothrombin time; RBC: red blood cell; TT: Thrombin time; WBC: white blood cell.

**Table S2** Different indicators between healthy adults and CVST patients.

| **Characteristics** | **Controls (n=48)** | **CVST (n=48)** | ***P* value** |
| --- | --- | --- | --- |
| WBC, ×10^9^/L | 6.93 ± 1.13 | 8.46 ± 2.47 | <0.001 |
| NEU, % | 56.84 ± 6.80 | 72.48 ± 8.87 | <0.001 |
| RBC, ×10^12^/L | 4.56 ± 0.51 | 4.54 ± 0.47 | 0.851 |
| HGB, g/L | 132.04 ± 11.41 | 131.83 ± 16.15 | 0.942 |
| PLT, ×10^9^/L | 219.65 ± 65.16 | 211.60 ± 64.73 | 0.446 |
| PT, s | 11.13 ± 0.63 | 10.90 ± 1.14 | 0.227 |
| APTT, s | 37.65 ± 2.77 | 31.33 ± 4.12 | <0.001 |
| TT, s | 17.20 ± 1.41 | 15.99 ± 1.64 | <0.01 |
| FIB, g/L | 3.05 ± 0.40 | 3.08 ± 1.06 | 0.881 |
| ADC, ×10^-3^ mm^2^/s | 0.76 ± 0.05 | 0.99 ± 0.24 | <0.001 |
| ICP, mmH_2_O | 130 ± 25.51^a^ | 218.33 ± 62.30 | <0.001 |

a: Normal range of CSF pressure for lumbar puncture (80–180 mmH_2_O).

ADC: apparent diffusion coefficient; APTT: activated partial thromboplastin time; CVST: cerebral venous sinus thrombosis; FIB: fibrinogen; HGB: hemoglobin; ICP: intracranial pressure; NEU: neutrophil; PLT: platelet; PT: prothrombin time; RBC: red blood cell; TT: Thrombin time; WBC: white blood cell.
